# Quantitative trait analysis of the development of pulmonary tolerance to inhaled zinc oxide in mice

**DOI:** 10.1186/1465-9921-6-73

**Published:** 2005-07-18

**Authors:** Scott C Wesselkamper, Lung Chi Chen, Terry Gordon

**Affiliations:** 1 Department of Environmental Health, University of Cincinnati Medical Center, Cincinnati, OH 45267, USA; 2 Department of Environmental Medicine, New York University School of Medicine, Tuxedo, NY 10987, USA

## Abstract

**Background:**

Individuals may develop tolerance to the induction of adverse pulmonary effects following repeated exposures to inhaled toxicants. Previously, we demonstrated that genetic background plays an important role in the development of pulmonary tolerance to inhaled zinc oxide (ZnO) in inbred mouse strains, as assessed by polymorphonuclear leukocytes (PMNs), macrophages, and total protein in bronchoalveolar lavage (BAL) phenotypes. The BALB/cByJ (CBy) and DBA/2J (D2) strains were identified as tolerant and non-tolerant, respectively. The present study was designed to identify candidate genes that control the development of pulmonary tolerance to inhaled ZnO.

**Methods:**

Genome-wide linkage analyses were performed on a CByD2F2 mouse cohort phenotyped for BAL protein, PMNs, and macrophages following 5 consecutive days of exposure to 1.0 mg/m^3 ^inhaled ZnO for 3 hours/day. A haplotype analysis was carried out to determine the contribution of each quantitative trait locus (QTL) and QTL combination to the overall BAL protein phenotype. Candidate genes were identified within each QTL interval using the positional candidate gene approach.

**Results:**

A significant quantitative trait locus (QTL) on chromosome 1, as well as suggestive QTLs on chromosomes 4 and 5, for the BAL protein phenotype, was established. Suggestive QTLs for the BAL PMN and macrophage phenotypes were also identified on chromosomes 1 and 5, respectively. Analysis of specific haplotypes supports the combined effect of three QTLs in the overall protein phenotype. Toll-like receptor 5 (*Tlr5*) was identified as an interesting candidate gene within the significant QTL for BAL protein on chromosome 1. Wild-derived *Tlr5*-mutant MOLF/Ei mice were tolerant to BAL protein following repeated ZnO exposure.

**Conclusion:**

Genetic background is an important influence in the acquisition of pulmonary tolerance to BAL protein, PMNs, and macrophages following ZnO exposure. Promising candidate genes exist within the identified QTL intervals that would be good targets for additional studies, including *Tlr5*. The implications of tolerance to health risks in humans are numerous, and this study furthers the understanding of gene-environment interactions that are likely to be important factors from person-to-person in regulating the development of pulmonary tolerance to inhaled toxicants.

## Introduction

Individuals may develop tolerance to adverse health effects elicited from repeated exposure to inhaled toxicants in several different occupational and environmental situations. Pulmonary tolerance can be defined as the lung's ability to withstand the detrimental effects of a toxic compound following multiple exposures. There are several implications of tolerance to human health, having both advantages and disadvantages with respect to the development of harmful health effects. Clinical investigations of zinc oxide (ZnO)- [1], endotoxin- [2], and ozone- [3-6] induced adverse respiratory effects have demonstrated inter-individual variability in the capacity to develop pulmonary tolerance following inhalation exposure. Because these clinical studies are more tightly controlled than epidemiologic studies, they suggest that genetic background and gene-environment interactions contribute to the development of pulmonary tolerance in humans.

We initially characterized the pulmonary tolerance phenotype in an outbred mouse model by assessing levels of BAL protein and polymorphonuclear leukocytes (PMNs) following single (1X) and 5 daily repeated (5X) exposures to inhaled ZnO to begin the identification of the genes regulating the development of pulmonary tolerance to repeated toxicant exposure [7]. Significant genetic variability in the development of pulmonary tolerance to ZnO, endotoxin, and ozone was established in several inbred strains of mice in a subsequent study [8]. Of the strains tested, the BALB/cByJ (CBy) strain was tolerant and the DBA/2J (D2) strain was non-tolerant to BAL protein, PMNs, and macrophages following repeated ZnO exposure.

Because inbred mouse strains are virtually identical at all loci throughout their genome, and also share several chromosomal regions of conserved synteny with humans, they are an ideal animal model in which to investigate genotype-environment interactions and identify genes controlling pulmonary responses to inhaled toxicants where no *a priori *evidence for their location exists [9]. Inbred strains of mice have been successfully utilized in quantitative trait locus (QTL) analyses to identify candidate genes that control susceptibility to adverse pulmonary responses induced by a variety of inhaled gases [10-13] and particulates [14,15]. In the present study, a CByD2F2 mouse cohort was used to determine QTLs linked to the development of pulmonary tolerance to BAL protein, PMN, and macrophage phenotypes following repeated ZnO exposure.

## Materials and methods

### Mice

Inbred BALB/cByJ (CBy), DBA/2J (D2) and CByD2F1/J (F1) mice (6–7 weeks of age) were purchased from The Jackson Laboratory (Bar Harbor, ME). CByD2F1/J mice were crossed to produce F2 (intercross) offspring in our laboratory animal facility. All mice were acclimated for at least 1 week before exposure, housed in a positive pressure environment with a 12-hour light/dark cycle starting at 6:00 a.m., and provided with water and standard laboratory rodent chow (Purina, Indianapolis, IN) *ad libitum *except during exposure. All mice were handled in accordance with the standards established by the U.S. Animal Welfare Acts set forth in National Institutes of Health guidelines, and by the New York University School of Medicine Division of Laboratory Animal Resources.

### Zinc oxide generation, characterization, and exposure

Mice were exposed to ZnO (1.0 ± 0.2 mg/m^3^, mean ± SD) in stainless steel cages placed inside a 0.07 m^3 ^Plexiglas chamber. ZnO fumes were generated in a furnace as previously described [7,16]. The ZnO particles had a mass median aerodynamic diameter of 0.3 μm and geometric standard deviation of 1.5. Samples of the chamber atmosphere were collected approximately every 40 minutes from the manifold of the exposure system with polytetrafluoroethylene filters (Type TX40HI20-WW, Pallflex Products Corp., Putnam, CT), and the ZnO concentration was determined gravimetrically using a microbalance (Model C-30, Cahn Instruments, Cerritos, CA).

For linkage analyses, all F2 offspring (*n *= 299, 138 males and 161 females) were exposed at 7–12 weeks of age in a total of seven 5X exposure regimens. Development of pulmonary tolerance was assessed 24 hours after the fifth exposure, and exposure group sizes ranged from 36 to 45 animals. All ZnO exposures of F2 mice also contained at least two CBy, D2, and F1 mice for control purposes.

### Bronchoalveolar lavage (BAL)

Mice were euthanized by intraperitoneal injections of ketamine HCl (100 mg/kg, Vetalar, Fort Dodge Laboratories, Inc., Fort Dodge, IA) and sodium pentobarbital (175 mg/kg, Sleepaway, Fort Dodge Laboratories, Inc.), and the posterior abdominal aorta was severed. The lungs of each mouse were lavaged two times with 1.2 ml of Dulbecco's phosphate buffered saline without Ca^2+ ^or Mg^2+ ^(pH 7.2–7.4, 37°C, Invitrogen, Carlsbad, CA). The collected BAL was immediately placed on ice (4°C) following recovery. Measurement of BAL protein, total cell counts, and differential cell counts were performed as previously described [8].

### DNA isolation and genotyping

Genomic DNA was isolated from kidney tissue of each phenotyped F2 animal and for CBy, D2, and F1 controls (Wizard Genomic DNA Purification Kit, Promega, Madison, WI). DNA concentration was determined using a Beckman DU-650 spectrophotometer, and each sample was diluted to 10 ng/μl for genotype analysis. PCRs were performed to genotype F2 offspring for SSLPs located throughout the mouse genome. Eighty-six unlabeled primer pairs for SSLPs that differed in length by at least 5% between the CBy and D2 progenitor strains were purchased from Research Genetics (ResGen/Invitrogen). PCR was performed in 20 μl reaction volumes in 96-well low profile plates (Fisherbrand, Fisher Scientific, Fairlawn, NJ) using a PTC-100 thermal cycler (MJ Research, Watertown, MA). The final concentration for each reaction was: 10 mM Tris-HCl (pH 8.3), 50 mM KCL, 2.5 mM MgCl_2_, 0.2 mM of each deoxynucleotide triphosphate (Promega), 1.1X Rediload (ResGen/Invitrogen), and 0.132 μM of each SSLP primer pair. This reaction mixture was added to 100 ng of genomic DNA and 0.45 U of *Taq *DNA polymerase (Roche Applied Science, Indianapolis, IN). Final reaction mixtures were initially denatured at 94°C for 3 min, followed by 36 amplification cycles (94°C for 30 seconds, 57°C for 45 seconds, and 72°C for 30 seconds + 1 second/cycle). A final extension step at 72°C for 7 min was followed by refrigeration (4°C). PCR products were differentiated on 3% agarose (Invitrogen) gels and all samples were visualized by ethidium bromide staining using a ChemiImager-4400 low light imaging system (Imgen Technologies, Alexandria, VA) and called by a single investigator. Any questionable calls in reading the genotype from the image were reviewed by a second investigator and if not resolved, that sample was rerun.

### Estimation of loci

The number of independently segregating loci was calculated using the following formula by Wright [17]: *n *= (P2 - F1)^2^/4(|σ^2^_F2 _- σ^2^_F1_|), where *n *is an estimate of the number of independent loci; P2 and F1 are the mean BAL protein responses following 5X ZnO exposure in CBy and CByD2F1 mice, respectively; σ^2^_F2 _and σ^2^_F1 _are the computed variances of the F2 and CByD2F1 mice, respectively.

### Linkage analyses

A genome scan was performed to identify associations between genotypes and the BAL protein phenotype using a CByD2F2 mouse cohort. All phenotypic data were natural log normalized to generate a normal distribution to meet normality assumptions of the Map Manager QT computer program. Interval analyses were then performed by fitting a regression equation for the effect of a theoretical QTL at the position of each SSLP and at 1-centimorgan (cM) intervals between SSLPs using free, additive, recessive, and dominant regression models. The regressions and significance of each genotype/phenotype association (or likelihood χ^2 ^statistic) were calculated by Map Manager [18]. Permutation tests were performed on the phenotypic and genotypic data using Map Manager to generate empirical thresholds for significance following the methods of Churchill and Doerge [19,20].

For the initial genome scan, the 15 most tolerant and 15 most non-tolerant F2 animals with respect to BAL protein, PMNs, and macrophages (i.e., the phenotypic extremes) were used for selective genotyping [9,21]. Interval analyses were done as stated previously, and 10,000 permutations were performed to generate significant and suggestive likelihood χ^2 ^statistic thresholds for the BAL protein phenotype. Following the identification of a suggestive QTL for BAL protein on chromosome 1, the entire F2 cohort was examined for additional QTLs. For the BAL protein phenotype, three additional SSLPs on chromosome 1 were analyzed, and a permutation test (10,000 permutations) was performed with only chromosome 1. This method was similar to that used in previous linkage studies with inhaled particles and gases that utilized Map Manager QT [10,14,22]. All likelihood χ^2 ^statistic thresholds corresponded to those reported in the aforementioned linkage studies.

### Haplotype analysis

A haplotype analysis was carried out similar to that done previously by Prows and Leikauf to determine the contribution of each QTL and QTL combination to the overall BAL protein phenotype [15]. This method quantifies any difference in mean BAL protein levels that are linked with a particular haplotype. Haplotypes for the following SSLPs were used for this analysis: *D1Mit291 *(101.5 cM), *D4Mit254 *(82.5 cM), and *D5Mit193 *(1.0 cM). Mean BAL protein concentrations for groups of F2 mice with the same haplotype at each QTL or QTL combinations were calculated and compared with the mean BAL protein of F2 mice with the other haplotypes to determine the contributions of these QTLs to the overall BAL protein phenotype.

## Results

### Phenotypes of the CByD2F2 cohort

**Figure 1:**
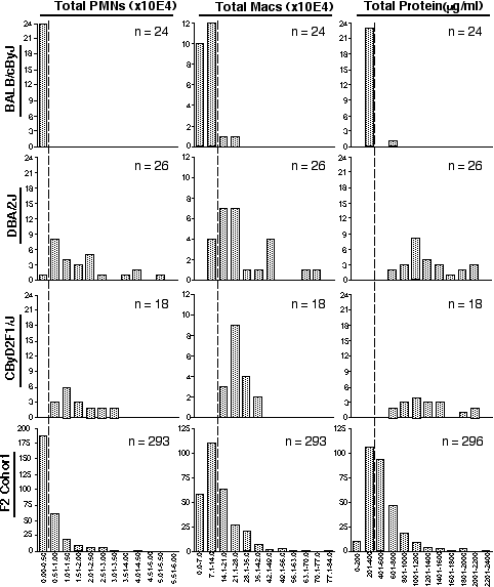
Frequency distribution of the number of BAL PMNs (×10^4^), macrophages (×10^4^), and protein (μg/ml) in BALB/cByJ, DBA/2J, CByD2F1/J, and CByD2F2 mice following 5 consecutive days of exposure to 1.0 mg/m^3 ^ZnO for 3 h/day. Vertical dashed lines represent approximate separation points between BALB/cByJ and DBA/2J phenotypes.

To further understand the role of genetic background in the development of pulmonary tolerance, an F2 (backcross) cohort derived from the CBy and D2 progenitors was phenotyped. The frequency distribution of the BAL protein, PMN, and macrophage phenotypic responses of the F2 cohort were within the ranges of similarly exposed CBy and D2 progenitor mice (Figure 1).

### Selective genotyping

**Figure 2:**
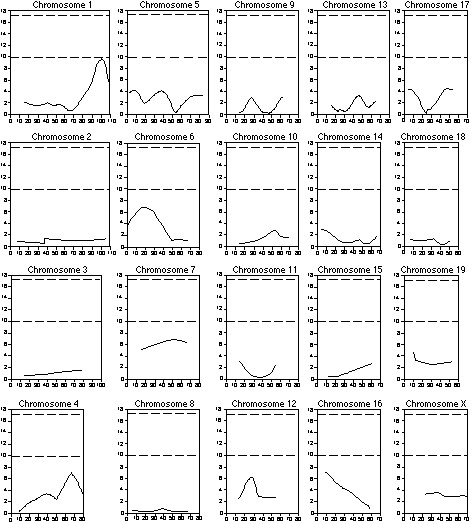
Genome-wide scan for QTLs associated with the BAL protein phenotype by selective genotyping of the CByD2F2 cohort. For each plot, the x-axis is the length of the chromosome in centimorgans (cM), and the y-axis is the likelihood χ^2 ^statistic value as calculated by Map Manager. The upper and lower dashed lines represent significant (LRS = 17.4) and suggestive (LRS = 9.9) linkage thresholds, respectively.

A genome-wide scan was performed using the 15 most tolerant and 15 most non-tolerant mice to initially identify possible QTLs influencing the development of pulmonary tolerance. Permutation tests on the BAL protein-extreme data set established a suggestive likelihood χ^2 ^statistic threshold of 9.9 and a significant likelihood χ^2 ^statistic threshold of 17.4. These values were consistent with the genome-wide probabilities projected by Lander and Kruglyak [23]. Interval mapping identified a suggestive QTL for the BAL protein phenotype on the distal end of chromosome 1 (Figure 2). No QTLs were identified for the PMN and macrophage phenotypes from selective genotyping.

### Genotyping of the entire F2 cohort

**Figure 3:**
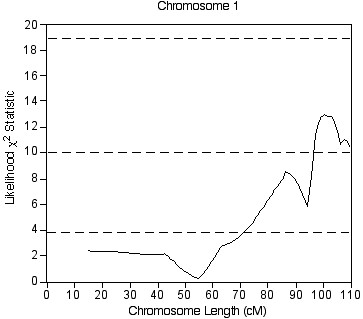
Plot of a significant QTL on chromosome 1 that is associated with the BAL protein phenotype from analysis of the entire CByD2F2 cohort. The x-axis is the length of the chromosome in centimorgans (cM), and the y-axis is the likelihood χ^2 ^statistic value as calculated by Map Manager. The lower dashed line represents the suggestive linkage threshold (LRS = 3.8), the middle dashed line represents significant linkage threshold (LRS = 10.0), and the upper dashed line represents the highly significant linkage threshold (LRS = 18.8).

The entire F2 cohort was genotyped with three additional SSLPs on distal chromosome 1 to further analyze the suggestive BAL protein QTL on chromosome 1 identified from selective genotyping. Interval mapping of the entire F2 cohort confirmed the QTL on chromosome 1 between 101.0 cM (*D1Mit426*) and 109.6 cM (*D1Mit293*) (Figure 3). The peak likelihood χ^2 ^statistic value for this QTL exceeded the threshold value of 10.0 for significant linkage as determined by 10,000 permutations with all loci from chromosome 1 only.

**Figure 4:**
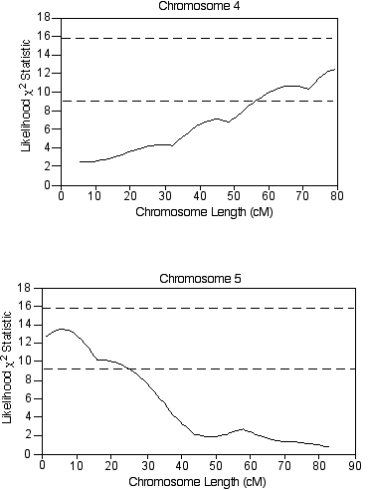
Plots of suggestive QTLs on chromosomes 4 and 5 associated with the BAL protein phenotype from analysis of the entire CByD2F2 cohort. The x-axis is the length of the chromosome in centimorgans (cM), and the y-axis is the likelihood χ^2 ^statistic as calculated by Map Manager. The upper and lower dashed lines in each plot represent significant (LRS = 15.8) and suggestive (LRS = 9.2) linkage thresholds, respectively.

**Figure 5:**
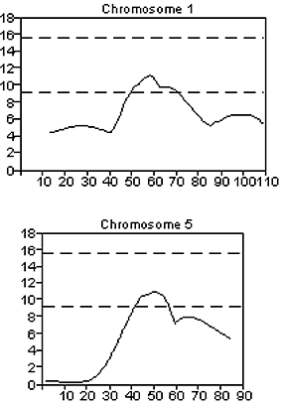
Plots of suggestive QTLs on chromosomes 1 and 5 associated with the BAL PMN and macrophage phenotypes, respectively, from analysis of the entire CByD2F2 cohort. The x-axis is the length of the chromosome in centimorgans (cM), and the y-axis is the likelihood χ^2 ^statistic as calculated by Map Manager. The upper and lower dashed lines in each plot represent significant (LRS = 15.6) and suggestive (LRS = 9.2) linkage thresholds, respectively.

The entire F2 cohort was further analyzed with the initial 86 SSLPs for the BAL protein, PMN, and macrophage phenotypes. Suggestive QTLs for the BAL protein phenotype that were not previously characterized by selective genotyping were identified on chromosome 4 between 53.6 cM (*D4Mit146*) and 82.5 cM (*D4Mit254*), and on chromosome 5 between 1.0 cM (*D5Mit193*) and 18.0 cM (*D5Mit148*) (Figure 4). Suggestive QTLs for the BAL PMN and macrophages were identified on chromosomes 1 and 5, respectively (Figure 5).

### Haplotype analysis

**Figure 6:**
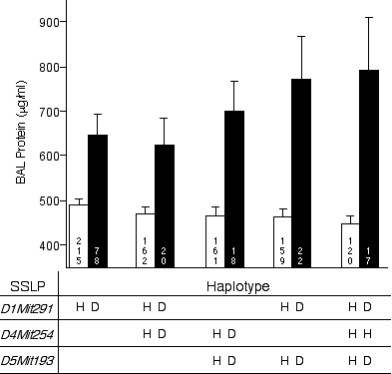
Differences in mean BAL protein levels of CByD2F2 mice with tolerant versus non-tolerant haplotypes at SSLPs representing the identified QTLs. *Open bars*, F2 mice with CC or CD genotypes (represented as H). *Filled bars*, F2 mice with a DD genotype (represented as D). *Number within each bar *is the number of F2 mice with the given genotype or haplotype. Values are means ± SE. All comparisons of tolerant (*open bars*) haplotypes versus non-tolerant haplotypes (*filled bars*) were significant (*P *< 0.05, *t *test).

Mean BAL protein levels of F2 mice with the same haplotype were calculated and compared with the mean BAL protein levels of F2 mice with the opposite haplotype in order to determine the contribution of each QTL and combinations of QTLs to the overall BAL protein phenotype (Figure 6). For each SSLP, F2 animals were genotyped as a homozygous CBy (CC), a homozygous D2 (DD) or heterozygous (H). For any individual SSLP, the greatest difference in mean BAL protein was found for *D1Mit291*. F2 mice that were DD at that locus had an average of 156 μg/ml more BAL protein than those mice that had CC or H haplotypes.

The combinatorial effect of the QTLs on chromosomes 1, 4, and 5 were also examined. For any combination of two QTLs, F2 mice that had a DD-DD haplotype for markers on chromosomes 1 and 5 (*D1Mit291 *and *D5Mit193*) had an average of 310 μg/ml more BAL protein than those that were CC or H (CC/H) for those markers. The greatest difference in mean BAL protein levels were found in F2 mice that had a DD-CC/H-DD haplotype for the three QTLs on chromosomes 1, 4, and 5, (i.e., *D1Mit291*, *D4Mit254*, and *D5Mit193*), respectively. These mice had an average of 345 μg/ml more BAL protein than those that were CC/H at the markers across the three chromosomes.

### Identification of candidate genes

Within all of the QTLs, candidate genes discovered with potential roles in controlling the development of tolerance to inhaled ZnO are presented in Tables 1 and 2. These genes were chosen as candidates from a thorough review of the existing literature and through the positional candidate gene approach, which combines knowledge of map position with the mouse transcript map [24].

**Table 1:** Positional candidate genes from linkage analysis of pulmonary tolerance to BAL protein following repeated ZnO exposure.

Chromosome	QTL Interval	Location	Human Orthology	Candidate Gene
Chr 1	92–112 cM	94.0 cM	1 (q21-q22)	*Dfy*, Duffy blood group
		98.0 cM	1 (q41-q42)	*Tlr5*, toll-like receptor 5
		98.6 cM	1 (q41-q42)	*Adprt1*, ADP-ribosyltransferase 1
		101.5 cM	1 (q41)	*Tgfb2*, transforming growth factor-β2
		105.0 cM	1 (q32)	*Traf5*, TNF receptor-associated factor 5
		106.0 cM	1 (q32-q41)	*Slc30a1*, solute carrier family 30 (zinc transporter), member 1
				
Chr 4	62–82 cM	62.4 cM	1 (p35-p34.3)	*Ptafr*, platelet-activating factor receptor
		65.7 cM	1 (p35.3)	*Slc30a2*, solute carrier family 30, member 2
		68.0 cM	1 (p35), 1 (p34-p36)	*Pla2g2a*, *2c*, *2d-f*, *5*, phospholipase A_2 _groups
		75.5	1 (p36.3-p36.2), 1 (p36)	*Tnfrsf1b*, *8*, *9*, TNF-receptor superfamily members
		79.4 cM	1 (p36)	*Tnfrsf4*, TNF-receptor superfamily, member 4
				
Chr 5	1–20 cM	14.0 cM	7 (q35-q36)	*Slc4a2*, solute carrier family 4, member 2
		17.0 cM	7 (p21)	*Il6*, interleukin 6
		18.0 cM	7 (pter-qter)	*Slc30a3*, solute carrier family 30 (zinc transporter), member 3

**Table 2:** Positional candidate genes from linkage analysis of pulmonary tolerance to BAL PMNs and macrophages following repeated ZnO exposure.

Chromosome	QTL Interval	Location	Human Orthology	Candidate Gene
Chr 1 (PMNs)	51–73 cM	52.0 cM	2 (q33-q37)	*Ccl20*, chemokine (C-C motif) ligand 20
		67.4 cM	2 (q21)	*Cxcr4 *chemokine (C-X-C motif) receptor 4
		69.9 cM	1 (q31-q32)	*Il10*, interleukin 10
				
Chr 5 (Macrophages)	43–58 cM	51.0 cM	4 (q13-q21)	*Areg*, amphiregulin
		51.0 cM	4 (q13-q21)	*Btc*, betacellulin
		51.0 cM	4 (q21)	*Cxcl1*, chemokine (C-X-C motif) ligand 1
		51.0 cM	4 (q21)	*Cxcl2*, chemokine (C-X-C motif) ligand 2
		53.0 cM	4 (q21)	*Cxcl5*, chemokine (C-X-C motif) ligand 5
		53.0 cM	4 (q21)	*Cxcl9*, chemokine (C-X-C motif) ligand 9
		53.0 cM	4 (q21)	*Cxcl10*, chemokine (C-X-C motif) ligand 10
		56.0 cM	4 (q21-q25)	*Spp1*, secreted phosphoprotein 1

### Development of pulmonary tolerance in MOLF/Ei mice

**Figure 7:**
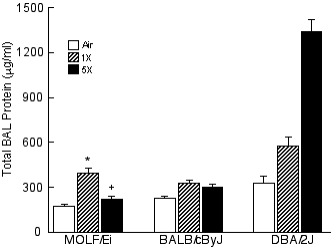
BAL protein levels in wild-derived MOLF/Ei mice 24 h after single (1X) or repeated (5X) exposure to 1.0 mg/m^3 ^ZnO or air for 3 h. Protein levels of BALB/cByJ and DBA/2J mouse strains following ZnO exposure are also shown for comparison purposes. Values are means ± SE (*n *= 4–5 MOLF/Ei mice/exposure group). * indicates significantly different from air-exposed MOLF/Ei controls, *P *< 0.05 [Student-Newman Keuls (SNK) test]. + indicates significantly different from 1X MOLF/Ei exposure group, *P *< 0.05 (SNK test).

We identified toll-like receptor 5 (*Tlr5*) as an interesting candidate gene within the significant QTL for BAL protein on chromosome 1. A wild-derived inbred mouse strain called MOLF/Ei (*M. m. molossinus*) which has non-conservative mutations in *Tlr5 *[25] was phenotyped for BAL protein following 1X and 5X ZnO exposure to determine whether *Tlr5 *may function in the development of pulmonary tolerance (Figure 7). MOLF/Ei BAL protein was significantly increased above control values following 1X ZnO exposure (395 ± 38 μg/ml). However, MOLF/Ei mice exhibited a tolerant phenotype, as 5X BAL protein values (215 ± 22 μg/ml) were significantly decreased below that of the 1X exposure group.

## Discussion

Clinical studies on the acquisition of tolerance to inhaled toxicants suggest that genetic background and gene-environment interactions contribute to the development of pulmonary tolerance in humans. We have previously determined that a genetic component exists in a mouse model of pulmonary tolerance to repeated ZnO exposure [8]. In the present study, we performed linkage analyses on an F2 mouse population derived from tolerant CBy and non-tolerant D2 strains to further ascertain the contribution of genetic background to the development of pulmonary tolerance, and identify candidate genes that may be important regulators in the acquisition of tolerance.

Initial analysis using the most tolerant and non-tolerant F2 mice with respect to BAL protein generated a putative QTL (designated as the zinc-induced tolerance (*ZIT*_1_) locus) on the distal end of chromosome 1. Further assessment of the entire F2 cohort demonstrated that the QTL on chromosome 1 attained an LRS value for significant linkage, and also identified two suggestive QTLs located on chromosomes 4 and 5. Using a variation of the Wright equation [17], a minimum of three loci were estimated to be independently segregating with the BAL protein phenotype following 5X ZnO exposure, thus in agreement with the results of the linkage analysis. There are several approaches that can be pursued to focus in on the QTL intervals that were identified. For instance, increasing the number of F2 mice used in the QTL analysis is one alternative. The major disadvantage of this, however, is that segregating QTLs contribute a great deal of phenotypic "noise," making it problematic when determining whether or not a given mouse has inherited a particular QTL [9]. Thus, in order to separate the effects of multiple loci, congenic mouse strains for each QTL could be generated, which could then be used to breed multicongenic lines to examine the existence of any epistatic effects. Additionally, future studies could employ the use of a backcross (CByD2F1 × CBy) mouse population to expand the evaluation of the QTL effects to the overall BAL protein phenotype.

To measure the contribution of each individual QTL and each QTL combination to the overall BAL protein phenotype, the protein levels for F2 mice with each particular haplotype were compared. Mice with opposite allelic combinations for QTLs on chromosomes 1 and 5 had a difference of 310 μg/ml BAL protein, which accounts for approximately one-third of the total difference in mean BAL protein between the parental CBy and D2 strains. Additionally, the mean BAL protein level of F2 mice with a DD haplotype for QTLs on chromosomes 1, 4, and 5 was over half that of the non-tolerant D2 parental strain. These analyses suggest that although three QTLs were identified, the development of pulmonary tolerance to BAL protein is a decidedly complex phenotype that is regulated by a number of different genes, some of which were likely not identified by linkage analysis using an F2 cohort.

Candidate genes within the significant QTL on chromosome 1 (*ZIT*_1_) that could play a role in controlling the development of tolerance to BAL protein following repeated ZnO exposure are presented in Table 1. The Duffy blood group (*Dfy*) has been shown to modulate the intensity of inflammation following endotoxin exposure [26], and has a role in enhancing inflammatory cell recruitment to sites of inflammation by facilitating movement of chemokines across the endothelium [27]. ADP-ribosyltransferase 1 (*Adprt1*) and tumor necrosis factor (TNF) receptor-associated factor (*Traf5*) are functionally associated with nuclear factor (NF)-κB, a key transcription factor in the regulation of the inflammatory process [28,29]. Additionally, activation of ADPRT1 plays a role in endotoxin-induced BAL protein increases [30,31]. Activated transforming growth factor-β has been shown to be a mediator of bleomycin- and endotoxin-induced lung permeability (i.e., BAL protein) in mice [32]. Finally, solute carrier family 30 (zinc transporter), member 1 (*Slc30a1*) is a metal transporter on the plasma membrane that confers resistance to zinc and cadmium toxicity *in vitro *via an efflux mechanism [33,34].

Candidate genes for BAL protein tolerance identified within the suggestive QTL intervals on chromosomes 4 and 5 are also presented in Table 1. Notable candidates include platelet-activating factor receptor (*Ptafr*) and phospholipase A_2 _(*Pla2*) that have been implicated as potential mediators of toxicant-induced BAL protein increases [35-37]. Interleukin (IL)-6 protein was increased following ZnO exposure in humans [1,38], and was hypothesized to be an anti-inflammatory suppressor of ZnO-induced lung injury. Interestingly, increased lung IL-6 levels have been shown to mediate pulmonary tolerance to ozone in rats [39]. Lastly, solute carrier family 30 (zinc transporter) members 2 and 3 (*Slc30a2 *and *Slc30a3*) have been identified as zinc transporters that protect against zinc-induced toxicity in both cell culture and animal models [40,41]. The development of pulmonary tolerance to BAL protein in our model could be regulated by any number of the aforementioned candidate genes. These candidates will be investigated in future studies of transcriptional and protein regulation to determine their roles in the development of tolerance.

The suggestive QTLs for tolerance to BAL PMNs and macrophages (on chromosomes 1 and 5, respectively) were also examined for positional candidate genes (Table 2). Chemokine (C-C motif) ligand 20 (*Ccl20*), chemokine (C-X-C motif) receptor 4 (*Cxcr4*), and interleukin-10 (*Il10*) were identified as candidates for BAL PMN tolerance. The movement of PMNs into inflammatory tissues is regulated by chemotactic factors (e.g., chemokines) that signal through numerous chemokine receptors [42]. PMNs are capable of producing several chemokines and proinflammatory cytokines, indicating that PMNs may be important in auto-direction of cell trafficking during inflammation. CXCR4 expression has been detected on human PMNs [43], and coordinated chemokine receptor gene expression may control the tissue-specific migration and activation status of PMNs into the lung. Human PMNs are also able to express CCL20 [44], which is able to recruit immature dendritic cells that play an important role in the initiation of the immune response as well as chronic inflammation [45-47]. Finally, IL-10 can be produced by T cells and is able to diminish PMN influx by inhibition of expression of proinflammatory chemokines [48], NF-κB (via I kappa kinase) [49], and TNF [50], as well as modulate cells and effector functions associated with an allergic response. With respect to the BAL macrophage phenotype, the epidermal growth factor receptor (EGFR) ligands amphiregulin (*Areg*) and betacellulin (*Btc*) were identified as candidate genes within the suggestive QTL on chromosome 5. Ligand-dependent activation of the EGFR by particles rich in metal content lead to activation of the MAP kinase signaling cascade and cytokine expression and secretion [51,52]. Secreted phosphoprotein 1 (*Spp1*, also known as osteopontin) was also identified, and it can act as a chemoattractant for macrophages [53,54]. Lastly, a host of chemokine (C-X-C motif) ligands were identified between 51 and 53 cM that could be involved in macrophage chemotaxis [55]. Again, tolerance to BAL PMNs and macrophages may be under the influence of any number of these candidate genes.

In the present study, we identified toll-like receptor (*Tlr5*) as a candidate gene within the significant *ZIT*_1 _QTL on chromosome 1 for tolerance to BAL protein. Toll-like receptors activate intra-cellular signaling that culminates in the induction of a multitude of effector genes [56]. *Tlr5 *has been shown to be an important gene in the immune response to Gram-positive and Gram-negative bacterial flagellin [57,58]. We utilized a wild-derived inbred mouse strain called MOLF/Ei which has non-conservative mutations in *Tlr5 *that are associated with a lower level of expression [25] to determine whether *Tlr5 *plays a role in the development of tolerance to BAL protein in our ZnO model. Because MOLF/Ei mice have a lower level of TLR5 mRNA expression compared to other strains [25], it was unclear whether we would observe tolerance after a single exposure to ZnO. Although the MOLF/Ei strain has no "wild-type" control strain per se, MOLF/Ei mice were tolerant to increased BAL protein following repeated ZnO exposure when compared to the non-tolerant D2 strain. These data support a role for *Tlr5 *in the development of tolerance to BAL protein. Interestingly, Kleeberger and colleagues identified *Tlr4 *as a candidate gene in a study of susceptibility to increased BAL protein in mice following a single exposure to 0.3 ppm ozone for 72 hours [22], which also suggests toll-like receptor signaling may be important in the regulation of inhaled toxicant-induced changes in BAL protein.

The mechanism through which *Tlr5 *signaling may regulate the development of tolerance to ZnO is unknown. While endotoxin [59] and bacterial flagellin [60] have been demonstrated as the primary ligands for *Tlr4 *and *Tlr5*, respectively, the ligand(s) that is responsible for toll-like receptor signaling following exposure to inhaled toxicants such as ozone and ZnO is unknown. Although there have been no studies on endogenous ligands for *Tlr5*, several endogenous ligands for *Tlr4 *have been identified. For example, fibronectin [61] and hyaluronic acid [62] are produced by lung cells during lung injury and are endogenous *Tlr4 *ligands. The downstream pathway through which *Tlr5 *may regulate the development of tolerance to ZnO-induced BAL protein is potentially via NF-κB, a transcription factor that is known to induce several cytokines involved in inhaled ZnO responsiveness such as IL-8 and IL-6 [1,63]. Furthermore, NF-κB-dependent gene expression is decreased in *Tlr5*-mediated tolerance to flagellin *in vitro *[64]. Thus, it is plausible that a mutation in *Tlr5*, a regulatory element upstream of NF-κB signaling, could modulate tolerance to BAL protein from repeated ZnO exposure. Finally, *Tlr5 *may function as a danger signal receptor in the development of ZnO tolerance, consistent with the "danger model" of innate immunity that explicates activation of the innate immune system by factors other than foreign antigens [65]. Whatever the case, much work is needed in understanding how toxicants function through toll-like receptor signaling mechanisms in the lung to regulate adverse responses such as BAL protein.

In summary, linkage analysis of a large F2 mouse cohort identified significant linkage of a QTL (*ZIT*_1_) on chromosome 1 associated with tolerance to BAL protein following repeated exposure to inhaled ZnO. Suggestive QTLs were also identified on chromosomes 4 and 5 for BAL protein, on chromosome 1 for BAL PMNs, and on chromosome 5 for BAL macrophages. Haplotype analysis suggested that the combinatorial effects of these three loci contributed to the overall phenotype, which agrees with the calculated number of segregating loci. *Tlr5 *was identified within the significant QTL for BAL protein on chromosome 1. Wild-derived *Tlr5*-mutant MOLF/Ei mice were determined to be tolerant to BAL protein following repeated ZnO exposure, suggesting a role for *Tlr5 *in the development of pulmonary tolerance to inhaled toxicants.

## Conclusion

These data substantiate genetic background as an important influence in the acquisition of pulmonary tolerance following exposure to inhaled toxicants such as ZnO, and promising candidate genes exist within the identified QTL intervals that would be good targets for additional studies on the pathogenesis of tolerance.

## Competing interests

The author(s) declare that they have no competing interests.

## Authors' contributions

SCW: Participated in the design and coordination of the study, performed the study, and drafted the manuscript.

LCC: Participated in the design of the study and helped draft the manuscript.

TG: Conceived the study, participated in the design and coordination of the study, and helped draft the manuscript.
